# PReS-FINAL-2066: Use of chiropractic care in danish children with juvenile idiopathic arthritis

**DOI:** 10.1186/1546-0096-11-S2-P78

**Published:** 2013-12-05

**Authors:** KS Kuhn, AJ Schou, AE Christensen

**Affiliations:** 1 HC Andersen Childrens Hospital, Odense, Denmark

## Introduction

Patients with musculoskeletal symptoms are often seeking chiropractic care. The use of chiropractic care among children with juvenile idiopathic arthritis (JIA) in Denmark is not known.

## Objectives

The objective of this study was to describe the use of chiropractic care in a cohort of children with JIA, and to describe a possible association to patient specific characteristics. It was evaluated, if the duration of symptoms before diagnosis was different among children who used chiropractic care compared to those who did not. It was evaluated whether the chiropractors played an active role in referral of children to the pediactric rheumatologist.

## Methods

A questionnaire survey among JIA patients and their parents from H. C. Andersen Children's Hospital in Odense, Denmark.

## Results

The study included 94 children and 86 responded (92%). Valid data were obtained in 83 children. Ten children (12%) received chiropractic care before they were diagnosed with JIA. The symptoms leading to chiropractic evaluation were neck pain (5 children, 50%), walking disability (3 children, 33%) and low back pain (1 child, 10%).

The mean duration of symptoms before diagnosis was (mean (SD)) 6.7 (4.4) months in the group seeking chiropractic care compared to 8.5 (10.4) in the remaining patients. The difference was statistically not significant (p = 0.34). Four children (5%) were seeking chiropractic care after they were diagnosed with JIA.

## Conclusion

A total of 12% of children with JIA in a Danish population-based cohort were seeking chiropractic care before the diagnosis of JIA. The main symptoms were neck pain and walking disabilities. The use of chiropractic care did not enhance the risk of delayed diagnosis, but the chiropractors were seldom the initiators of a referral. Few patients used chiropractic care after being diagnosed and their main complain was low back pain.

## Disclosure of interest

None declared.

